# Niraparib promotes ferroptosis by inhibiting TM4SF1 expression through ALKBH1-mediated 6mA modification in BRCA wild-type ovarian cancer

**DOI:** 10.3389/fphar.2026.1706364

**Published:** 2026-06-01

**Authors:** Po-Wu Liu, Zhao-Yi Liu, Nayiyuan Wu, Xiu Zhang, Jia-Jia Sheng, Sheng-An Zheng, He Li

**Affiliations:** 1 The Affiliated Cancer Hospital of Xiangya School of Medicine, Central South University/Hunan Cancer Hospital, Changsha, Hunan, China; 2 Department of Obstetrics and Gynecology, Foshan Sanshui District People’s Hospital (Sanshui Hospital, Zhujiang Hospital, Southern Medical University), Foshan, Guangdong, China; 3 Hunan Provincial Key Laboratory of the Research and Development of Novel Pharmaceutical Preparations, the “Double-First Class” Application Characteristic Discipline of Hunan Province (Pharmaceutical Science), Changsha Medical University, Changsha, Hunan, China

**Keywords:** ALKBH1, ferroptosis, N6-methyladenine modification, niraparib, ovarian cancer, TM4SF1

## Abstract

**Objective:**

Niraparib significantly improves the prognosis of patients with BRCA wild-type ovarian cancer (BRCAwt OC). However, the underlying mechanisms remain elusive. N6-methyladenine DNA (6mA) modification has emerged as a critical epigenetic regulator in cancer progression. We investigated whether niraparib exerts antitumor effects by modulating 6mA modification in BRCAwt OC models.

**Methods:**

The 6mA modification levels were assessed using dot blot, ELISA, and 6mA-IP-seq. The regulatory role of ALKBH1 in transmembrane 4 L six family member 1 (TM4SF1) mRNA expression and 6mA modification was examined via RT-qPCR, Western blot, and 6mA-IP-qPCR, respectively. Using the CCK-8 assay, colony formation assay, wound healing assay, Transwell assay, and subcutaneous tumor xenograft models, the function of TM4SF1 in tumor growth was evaluated *in vivo* and *in vitro*. Additionally, the association between TM4SF1 expression and ferroptosis was assessed by measuring Fe^2+^ and lipid reactive oxygen species (ROS) levels using a transmission electron microscope (TEM).

**Results:**

Our findings demonstrated that niraparib treatment significantly reduced the expression of TM4SF1 by increasing ALKBH1-mediated 6mA modification. Mechanistically, niraparib increases the expression of ALKBH1, which binds to the TM4SF1 promoter, thereby regulating its 6mA modification and suppressing its expression. Furthermore, TM4SF1 knockdown reduced cell proliferation, invasion, and migration, along with tumor growth *in vivo* and *in vitro*. Inhibition of TM4SF1 enhanced Fe^2+^ accumulation and lipid ROS production, leading to the induction of ferroptosis.

**Conclusion:**

Niraparib exhibits antitumor effects and promotes ferroptosis by inhibiting TM4SF1 expression through ALKBH1-mediated 6mA modification in BRCAwt OC. These findings emphasize the potential application of niraparib in BRCAwt OC and reveal the important role of epigenetic regulation in cancer treatment.

## Introduction

Ovarian cancer (OC) is the third most common and deadliest gynecologic malignant tumor worldwide (Richardson et al., 2023). In 2024, approximately 19,680 newly diagnosed OC cases and 12,740 cancer deaths are estimated in the United States (Siegel et al., 2024). The lack of early diagnostic biomarkers often leads to advanced-stage diagnosis, resulting in poor outcomes for most patients (Siegel et al., 2023). Thus, improving early diagnosis and treatment strategies for OC remains a pressing clinical challenge. The introduction of poly (ADP-ribose) polymerase inhibitors (PARPis) has marked a significant milestone in the treatment and management of OC. With the approval of PARPi maintenance therapy in clinical practice, the 5-year survival rate has gradually improved over the past few decades. Additionally, the traditional treatment paradigm—surgery, chemotherapy, relapse, and post-recovery treatment—has evolved into a “new” treatment paradigm: surgery, chemotherapy-maintenance therapy, relapse, post-recovery treatment, maintenance therapy, recurrence, and post-recurrence therapy (Siegel et al., 2023). OC patients harboring homologous recombination repair defects (HRDs) or BRCA1/2 mutations (BRCAm) particularly benefit from PARPis. These genetic alterations are highly prevalent in OC. In these individuals, PARPis kill tumor cells primarily through “synthetic lethality.” Specifically, PARP1/2 plays a critical role in the process of DNA single-strand break repair (SSBR). Inactivation of PARP1/2 disrupts both SSBR and homologous recombination repair (HRR) in patients with BRCAm or HRD, leading to genome instability and cell death (Bétermier et al., 2014; Nielsen et al., 2016). Niraparib, as a type of PARPi, has been approved for the maintenance treatment of patients with newly diagnosed or relapsed OC (Lord and Ashworth, 2017). Recently, the results of the NOVA (ENGOT-OV16) trial suggested that niraparib remarkably prolonged progression-free survival (PFS) in OC patients, regardless of BRCA and HRD status (Tew et al., 2020). This suggests that niraparib may exhibit additional antitumor mechanisms beyond “synthetic lethality” in BRCA wild-type ovarian cancer (BRCAwt OC).

The 6mA modification is a DNA methyltransferase-catalyzed modification that adds a methyl group at position 6 of the adenine ring. Several genes, including ALKBH1, ALKBH4, N6AMT1, and METTL4, have identified as the key regulators of 6mA modification (Li et al., 2020). 6mA modification sites are commonly enriched in the coding regions of exons, marking transcriptionally active genes (González-Martín et al., 2019). Accumulating evidence highlights the critical role of 6mA modification in diverse biological processes including gene expression (Wion and Casadesús, 2006; Luo et al., 2015). Although 6mA modification is well characterized in prokaryotes, it has also been recently discovered in higher eukaryotes, including mammals (Greer et al., 2015; Xiao et al., 2018). Furthermore, abnormal 6mA modification has been reported to contribute to various diseases, including neurological disorders, hypertension, and various malignancies (Zhang et al., 2015). Importantly, the regulation pattern of 6mA modification may vary across different cancer types. For instance, 6mA modification is highly upregulated in esophageal squamous cell carcinoma and glioblastoma, whereas it is decreased in primary gastric cancer, hepatocellular carcinoma, and lung cancer (Wu et al., 2016; Xie et al., 2018). At present, the role of 6mA modification in OC prognosis and its underlying mechanisms remain poorly understood.

Transmembrane 4 L six family member 1 (TM4SF1) is a transmembrane protein that is composed of intracellular and extracellular domains (Liang et al., 2016). Multiple studies have demonstrated that TM4SF1 acts as a pro-oncogene and may serve as a tumor-associated antigen. Compared to normal tissues, TM4SF1 is abnormally highly expressed in various types of epithelial cancers, including pancreatic cancer, hepatocellular carcinoma, lung cancer, bladder cancer, and prostate cancer (Wright et al., 2000). Moreover, its high expression indicates a poor prognosis for cancer patients (Chang et al., 2005; Shih et al., 2009; Cao et al., 2016; Xue et al., 2017). Preclinical studies have revealed that TM4SF1 serves as an oncogene and promotes cancer cell proliferation, invasion, and metastasis; conversely, its inhibition significantly reduces the metastatic ability of cancer cells (Allioli et al., 2011; Zukauskas et al., 2011; Huang et al., 2016; Yang et al., 2017).

As a form of programmed cell death, ferroptosis is primarily regulated by iron ions and lipid metabolism, which are the main hallmark features of ferroptosis (Stockwell et al., 2017). GPX4, HSPB1, and SLC7A11 are important ferroptosis-related biomarkers, and they regulate ferroptosis through iron metabolism and lipid peroxidation pathways (Wang et al., 2019). Accumulating evidence has shown that ferroptosis participates in the progression of various diseases, including cancers, stroke, and ischemia–reperfusion injury in mammals (Jiang et al., 2021). Recent studies have highlighted the pivotal role of ferroptosis in inhibiting tumor growth, offering a promising avenue for cancer therapy (Xie et al., 2016).

In our study, we first demonstrated that niraparib significantly reduced the 6mA modification levels by upregulating ALKBH1 expression. ALKBH1 binds to the promoter of TM4SF1 and regulates its expression in a 6mA-dependent manner. Specifically, inhibition of TM4SF1 expression suppressed cell proliferation, invasion, and migration and induced ferroptosis. In conclusion, our findings suggested that niraparib exhibited its antitumor effects and promoted ferroptosis by inhibiting TM4SF1 expression through ALKBH1-mediated 6mA modification in BRCAwt OC.

## Materials and methods

### Cancer cell culture

Human BRCAwt OC cells SKOV3 and OVCAR8 were provided by the Institute of Clinical Pharmacology, Central South University. Both cell lines were cultured in RPMI-1640 medium (Gibco, United States) supplemented with 10% fetal bovine serum (Gibco, United States) and 1% penicillin/streptomycin (Gibco, United States) at 37 °C with 5% CO_2_.

### CCK-8 assay

Cells at the logarithmic growth phase were seeded into 96-well plates at a density of 5 × 10^3^ cells/well. After 12 h of incubation, they were treated with a series of niraparib concentrations (Selleck, United States). Sequentially, the CCK-8 reagent (ZETA, China) was added at 10 μL/well and incubated with the cells at 37 °C for 4 h. The optical density (OD) values at 450 nm were measured (Hong et al., 2021).

### Clone formation assay

Cells were transfected with siTM4SF1 or siNC as a negative control. After 24 h of transfection, cells were seeded at a density of 1,000 cells per well in 6-well culture plates and incubated for 1–2 weeks to allow colony formation. Finally, colonies were stained with crystal violet and counted using ImageJ software.

### Dot blot assay

Total DNA was extracted from cells using the genomic DNA purification kit (Omega, United States). The samples were denatured and spotted onto nitrocellulose membranes. The membrane was baked at 65 °C and blocked with goat serum. Thereafter, the membrane was incubated with the 6mA antibody (1:1000; Synaptic Systems, United States) overnight at 4 °C. The membrane was washed with PBST and incubated with a 1:4,000 dilution of the HRP-conjugated anti-rabbit IgG secondary antibody (1:4,000; Abcam, United States). Finally, the membranes were washed and treated with enhanced chemiluminescence (Zhang et al., 2015).

### ELISA

Cells were treated with 50 µM niraparib or DMSO. After incubation for 48 h, total DNA was extracted, and 6mA modification was measured using a fluorometric analysis kit (Epigentek, United States), according to the manufacturer’s instructions. In brief, the methylated fraction of gDNA was bound to a strip well using DNA high-binding solutions and recognized using m6A antibodies. Then, the samples were analyzed using a plate reader by measuring the OD value at 405 nm (D'Aquila et al., 2023).

### 6mA-IP-seq

The purified gDNA was treated with RNase and fragmented into 200–500 bp. The fragmented gDNA was immunoprecipitated with the rabbit 6mA antibody (Synaptic Systems, United States) at 4 °C for 2 h. Then, the samples were incubated with protein A/G Agarose beads at 4 °C for 2 h and washed with immunoprecipitation buffer. Subsequently, the beads were collected and treated with proteinase K. The methylated DNA was sequenced and analyzed by LC-Bio technology Co., Ltd. (Hangzhou, China).

### Western blot

Western blot was performed as described (Liu et al., 2019; Xia et al., 2020). In brief, proteins were extracted using RIPA lysis buffer (Beyotime, China). After extraction, the protein concentration was detected using a BCA assay kit (Thermo Fisher, United States). Subsequently, the proteins were loaded to SDS-PAGE, transferred onto a PVDF membrane, and blocked in 5% nonfat milk. After that, membranes were successively incubated with primary and secondary antibodies. After washing with TBST, they were used to measure the expression of indicated proteins. The primary antibody concentrations used for Western blotting were ALKBH1 (1:1,000, Abcam, United States), TM4SF1 (1:2,000, Sigma, United States), and GAPDH (1:1,000, Proteintech, United States). Finally, the relative protein expression was calculated using ImageJ software.

### RT-qPCR

Total RNA was extracted from cells using TRIzol reagent and reverse-transcribed into cDNA using the PrimeScript RT Reagent Kit (TaKaRa, Dalian, China). SYBR Premix Ex Taq II (TaKaRa, Dalian, China) was used for RT-qPCR on the Roche LightCycler® 96 instrument. The threshold cycle (Ct) values of the indicated genes were normalized to the housekeeper gene GAPDH. The relative expression of the indicated genes was analyzed and calculated using the 2^−ΔΔCt^ method. The primer sequences used in this study are listed in Supplementary Table S1.

### 6mA-IP-qPCR

DNA was extracted using the genomic DNA purification kit (Omega, United States) and sonicated into 200–500 bp fragments. The fragmented gDNA was incubated with the anti-6mA antibody (Synaptic Systems, United States) at 4 °C for 2 h. The mixture was then immunoprecipitated using protein A/G agarose beads at 4 °C for 2 h. After washing, the beads were treated with proteinase K, and the bound DNA was eluted from the elution buffer at 65 °C. Finally, the purified fold-enrichment of the fragment was measured using quantitative real-time PCR (Xiao et al., 2018).

### CHIP-qPCR

CHIP-qPCR was performed using the CHIP Assay Kit (Beyotime, China) according to the manufacturer’s instructions. In brief, cells were cross-linked with 1% formaldehyde and quenched by glycine solution. Subsequently, cells were lysed and sonicated. The cell lysates were incubated with the ALKBH1 antibody or the IgG antibody at 4 °C overnight. Then, protein A/G agarose beads were added to the mixture and rotated at 4 °C for another 1 h. After washing, DNA fragments were eluted, purified, and used for real-time PCR (Teng et al., 2021).

### Wound healing assay

Cells were incubated in 6-well plates. When the confluency reached approximately 90%, the cells were wounded with pasteurized pipette tips and cultured for 48 h in RPMI-1640 medium without fetal bovine serum at 37 °C with 5% CO_2_. The remaining wounded areas were photographed and measured.

### Transwell assay

Cells were seeded into the upper Transwell chambers without FBS. RPMI-1640 supplemented with 10% FBS was pre-coated in the lower chamber. The cells were incubated at 37 °C with 5% CO_2_ for 48 h. The upper chambers were rinsed with phosphate-buffered saline (PBS) several times. The migrated cells were fixed with 4% paraformaldehyde and stained with crystal violet. Finally, the number of migrated cells was counted under a microscope by selecting five different fields.

### Fe^2+^ assay

The intracellular Fe^2+^ density was measured using the Iron Assay Kit (Dojindo, China). According to the protocol, cells from different groups were cultured in a 12-well plate at 37 °C with 5% CO_2_ overnight. Subsequently, cells were washed with HBSS and stained with 1 μmol/L FerroOrange in the incubator for 30 min. The fluorescence of the cells at 543 nm/580 nm was analyzed using fluorescence microscopy (Xuan et al., 2022).

### BODIPY 581/591 C11 assay

Lipid ROS activity was detected using the BODIPY 581/591 C11 Assay Kit (Beyotime, China) according to the protocol. In brief, cells were seeded and cultured in a laser confocal culture dish at 37 °C with 5% CO_2_ overnight. After treating with vehicle or ferrostatin-1 (5 μM) for 24 h, cells were incubated and stained with the BODIPY 581/591 C11 reagent in the incubator for 30 min. Finally, the fluorescence of cells was analyzed using confocal laser scanning microscopy (Wu et al., 2024).

### Transmission electron microscope

Cells were fixed with 2.5% glutaraldehyde for 24 h. Subsequently, the cells were successively treated with 0.1% cacodylate-buffered tannic acid, 1% buffered osmium, and 1% uranyl acetate. After dehydration and embedding, cells were incubated at 60 °C for 24 h and photographed using a transmission electron microscope (TEM, HITACHI, Tokyo, Japan).

### *In vivo* experiment

Six-week-old, female BALB/c nude mice were purchased from SLA Laboratory Animal (Changsha, China). Following a week-long acclimation period, mice were subcutaneously injected with shNC or shTM4SF1 SKOV3 cells (2 × 10^6^ cell/mouse) into the left armpit region. After 4 weeks, the mice were sacrificed via cervical dislocation after anesthesia, and the xenograft tumors were collected and weighed. The experiments were approved by the Animal Care Committee of Hunan Cancer Hospital and the Affiliated Cancer Hospital of Xiangya School of Medicine (Hong et al., 2021).

### Statistical analysis

All the data from this study are presented as the mean ± SD from at least five independent experiments. SPSS 25.0 statistical software was used for statistical processing. Differences between two groups were compared using the Mann–Whitney U test. Overall survival and progression-free survival were analyzed using the Kaplan–Meier method and log-rank test. *p < 0.05; **p < 0.01; ***p < 0.001; n.s indicates non-significant.

## Results

### Niraparib reduced the 6mA modification by increasing the ALKBH1 expression in BRCAwt OC cell lines

**FIGURE 1:**
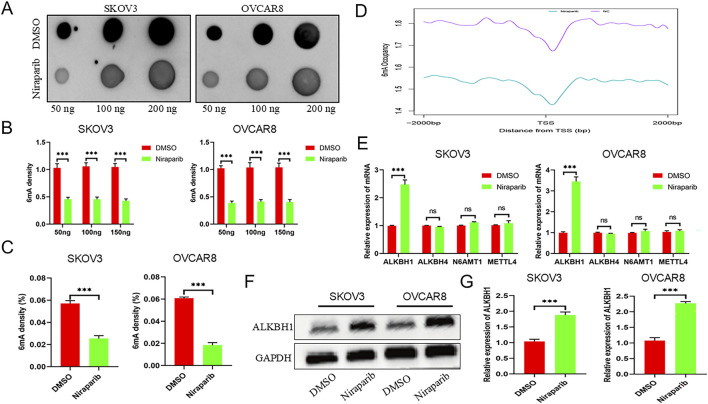
Niraparib treatment significantly reduced the 6mA modification level by increasing ALKBH1 expression in SKOV3 and OVCAR8 cells. **(A)** Dot blot assay to assess the influence of niraparib on genome 6mA modification. **(B)** Quantitative analysis result of the dot blot assay. **(C)** ELISA analysis to measure the 6mA modification level. **(D)** 6mA-IP-seq result. **(E)** The mRNA expressions of ALKBH1, N6AMT1, METTL4, and ALKBH4 in SKOV3 and OVCAR8 cell lines. **(F)** Expression of the ALKBH1 protein detected using Western blot. **(G)** Quantitative analysis result of Western blot. Data are presented as the means ± SD. ***P < 0.001, vs. DMSO group.

We treated BRCAwt OC cells (SKOV3 and OVCAR8) with a series of niraparib concentration and found that the IC_50_ values were 68.73 µM and 46.95 µM, respectively (Supplementary Figure S1). For subsequent experiments, cells were treated with 50 µM of niraparib. Compared with the DMSO group, dot blot analysis and ELISA revealed a significant decrease in 6mA modification levels in SKOV3 and OVCAR8 cells following niraparib treatment (Figures 1A–C). Furthermore, the 6mA-IP-seq analysis was performed in the SKOV3 cell line, and the 6mA-IP-seq result is consistent with the dot blot analysis and ELISA (Figure 1D). So far, several 6mA methyltransferases and demethylases, including ALKBH1, ALKBH4, N6AMT1, and METTL4, have been identified to participate in 6mA modification [9]. Among them, niraparib significantly upregulated ALKBH1 expression (Figures 1E–G). These results indicated that niraparib reduced 6mA modification by increasing ALKBH1 expression in BRCAwt OC cell lines. Additionally, compared with normal ovarian tissues, ALKBH1 was significantly downregulated in OC tissues, and low ALKBH1 expression was marginally associated with worse overall survival in the TCGA and GEO datasets (Supplementary Figure S2A,B). Furthermore, our results demonstrated that the combination with compound 13h (a selective small-molecule inhibitor of ALKBH1) might reduce the niraparib sensitivity of SKOV3 and OVCAR8 cells (Supplementary Figure S2C).

### Niraparib downregulated TM4SF1 expression by ALKBH1-mediated 6mA modification

**FIGURE 2:**
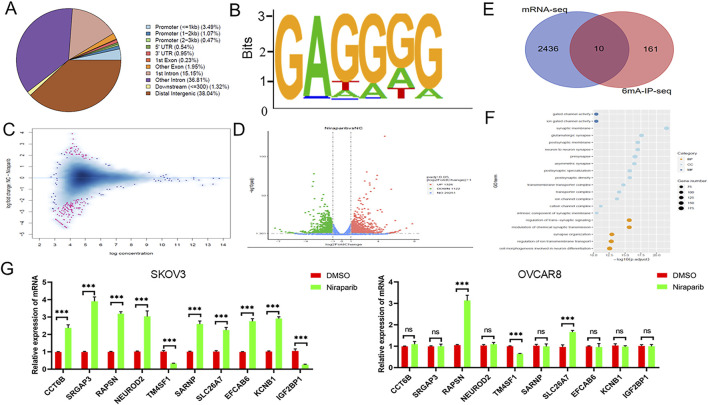
Effect of niraparib on 6mA modifications and mRNA expression in SKOV3 and OVCAR8 cells. **(A)** The distribution of 6mA modifications in the genome of SKOV3. **(B)** Motif of 6mA obtained by sequencing. **(C)** Differential 6mA modification of genes after treating with niraparib. **(D)** Volcano plot of differentially expressed genes (DEGs) after treating with niraparib. **(E)** Venn diagram of gene intersection between differentially 6mA modificatory genes and DEGs. **(F)** Enrichment analysis to assay the pathways affected by niraparib. **(G)** mRNA expression of indicated genes in SKOV3 and OVCAR8 cell lines. ***p < 0.001, n.s indicates non-significant vs. DMSO group.

**FIGURE 3:**
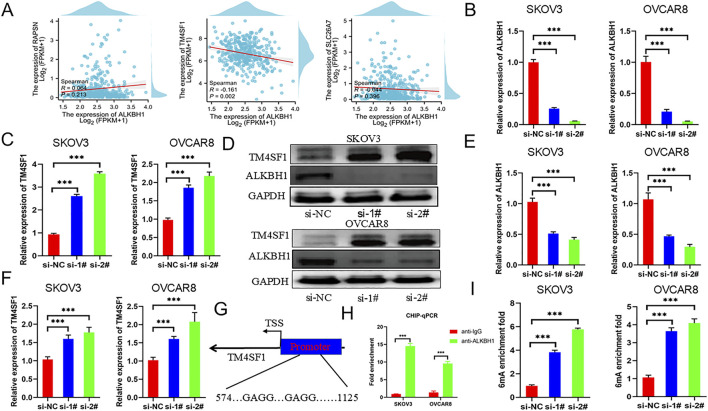
ALKBH1 suppressed TM4SF1 expression by binding to its promoter to mediate its 6mA modification in SKOV3 and OVCAR8 cells. **(A)** Correlation between ALKBH1 and RAPSN, along with TM4SF1 and SLC26A7, in the TCGA dataset. mRNA expression of ALKBH1 **(B)** and TM4SF1 **(C)** after ALKBH1 inhibition. **(D)** Protein expressions of ALKBH1 and TM4SF1 after ALKBH1 inhibition. **(E,F)** Quantitative analysis result of WB. **(G)** 6mA modification site in TM4SF1. **(H)** CHIP-qPCR results suggested that ALKBH1 bound to the TM4SF1 promoter. **(I)** Effect of ALKBH1 inhibition on 6mA modification in the TM4SF1 promoter measured using 6mA-IP-qPCR. Data are presented as the means ± SD. ***p < 0.001 vs. si-NC group.

Mapping the distribution of 6mA modifications across the genome is crucial for understanding its regulatory role. As shown in Figure 2A, most of the 6mA modification sites were predominantly enriched in intergenic regions, followed by the intronic and promoter regions in SKOV3 cells. Chromosomal enrichment analysis showed a wide range of enrichment in autosomes, while 6mA peaks in mitochondria and the Y chromosome were less abundant (Supplementary Figure S3A). Motif identification revealed a major motif of GAG[T/A]G[A]G (Figure 2B). Given the association between 6mA modification and gene transcription (Bergman and Cedar, 2013), we performed RNA-seq and 6mA-IP-seq analyses to identify genes whose expression is regulated by niraparib via 6mA modification. Using log_2_Foldchange ≥ 1 and P_adj_ < 0.05 as criteria, the expression of 2,436 genes was regulated by niraparib, and 161 genes with 6mA-modified alterations were obtained by 6mA-IP-seq (Figures 2C,D; Supplementary Figure S3B; Supplementary Tables S2-S3). Among these genes, only 10 genes intersected, indicating that their expression was regulated by niraparib through 6mA modification (Figure 2E; Supplementary Table S4). GO enrichment analysis revealed that the differential 6mA peaks were mainly enriched in gated channel activity, ion-gated channel activity, and synaptic membrane activity (Figure 2F). Furthermore, we validated the regulatory effect of niraparib on these 10 genes. The results showed that niraparib upregulated RAPSN and SLC26A7 mRNA expressions and downregulated TM4SF1 mRNA expression in OVCAR8 cells, which is consistent with the SKOV3 cell line (Figure 2G). To explore whether niraparib regulates these three genes via ALKBH1-mediated 6mA modification, we first analyzed the correlation between ALKBH1 and the three genes in the TCGA dataset. We found that the expression of ALKBH1 was negatively correlated with TM4SF1 expression, but there was no association between ALKBH1 and RAPSN or SLC26A7 (Figure 3A). Subsequently, we inhibited the expression of ALKBH1 using RNAi (Figures 3B,D,E). Our results suggested that ALKBH1 inhibition increased the expression of TM4SF1 mRNA and protein (Figures 3C,D,F). Moreover, treating with compound 13h also increased the mRNA expression of TM4SF1 (Supplementary Figure S4). 6mA-IP-seq results further demonstrated that the decreased 6mA modification in TM4SF1 was primarily enriched in the promoter region (Figure 3G). To validate that ALKBH1 regulates TM4SF1 expression by directly binding to its promoter and regulating 6mA modification, we first performed CHIP-qPCR, and the results suggested that ALKBH1 could directly bind to the promoter of TM4SF1 (Figure 3H). Subsequently, the 6mA-IP-qPCR results confirmed that ALKBH1 inhibition increased the 6mA modification levels of TM4SF1 (Figure 3I). Collectively, our results demonstrated that niraparib might downregulate the expression of TM4SF1 through ALKBH1-mediated 6mA modification.

### Downregulation of TM4SF1 inhibited cell proliferation, invasion, and metastasis *in vitro* and *in vivo*

**FIGURE 4:**
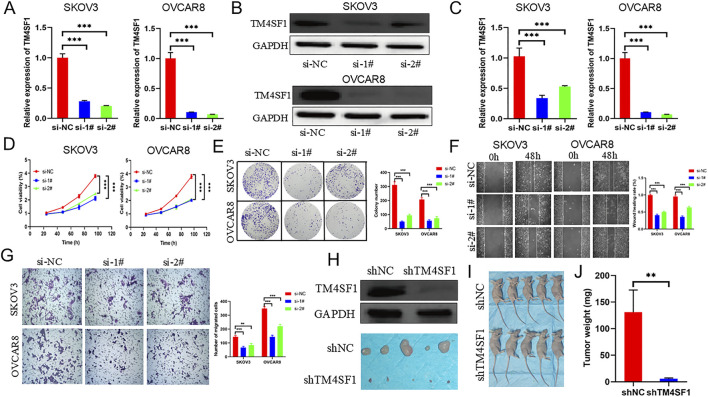
TM4SF1 inhibition suppressed cell proliferation, invasion, and tumor growth in SKOV3 and OVCAR8 cells. **(A,B)** Expressions of TM4SF1 mRNA and protein after TM4SF1 inhibition in SKOV3 and OVCAR8 cells. **(C)** Quantitative analysis result of WB. **(D)** CCK-8 assay to measure cell viability after TM4SF1 knockdown. **(E)** Representative images of the clone formation assay. **(F)** Representative images and the quantitative analysis result of the wound healing assay. **(G)** Representative images and the quantitative analysis result of the Transwell assay. **(H)** Establishment of a SKOV3 cell with TM4SF1 knockdown. **(I,J)** Representative images of tumor issues in the xenograft mouse model. **(K)** Effect of TM4SF1 knockdown on tumor weight in the xenograft mouse model. Data are presented as the means ± SD. **p < 0.01 and ***p < 0.001 vs. si-NC group or shNC group.

To explore the antitumor effects of niraparib by decreasing TM4SF1 expression, we further explored the association between TM4SF1 expression and ovarian cancer prognosis. Undoubtedly, TM4SF1 mRNA expression was significantly higher in ovarian tumor tissues than in normal ovary tissues (Supplementary Figure S5A). Kaplan–Meier plotter analysis found that lower TM4SF1 mRNA expression was remarkably associated with better clinical outcomes (Supplementary Figure S5B). To further explore the oncogenic effect of TM4SF1, TM4SF1 was knocked down in SKOV3 and OVCAR8 cells (Figures 4A–C). TM4SF1 knockdown significantly inhibited cell proliferation, invasion, and migration (Figures 4D–G). To further investigate the effects of TM4SF1 on tumorigenesis *in vivo*, we established a SKOV3 cell line with stable TM4SF1 knockdown and inoculated it into BALB/c nude mice (Figure 4H). Compared to the control group, inhibition of TM4SF1 significantly reduced tumor growth (Figures 4I–K). These findings demonstrated that downregulation of TM4SF1 effectively inhibited cell proliferation and tumor growth *in vitro* and *in vivo*.

### TM4SF1 inhibits ferroptosis in BRCAwt OC cell lines

**FIGURE 5:**
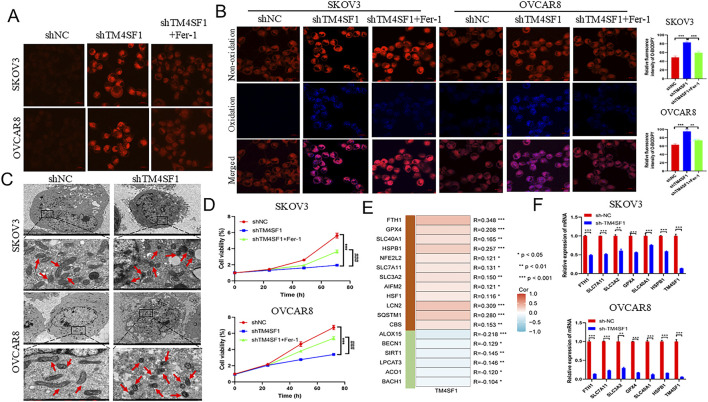
TM4SF1 inhibition induced ferroptosis in SKOV3 and OVCAR8 cells. **(A,B)** Representative images of the density of Fe^2+^ **(A)** and lipid ROS **(B)** in SKOV3 and OVCAR8 cells (scale bar = 20 µm). **(C)** Representative images of TEM (scale bar = 500 nm). **(D)** Fer-1 significantly rescued the inhibition of cell proliferation induced by TM4SF1 knockdown. **(E)** Correlation between TM4SF1 and indicated ferroptosis-related genes in the TCGA dataset. **(F)** mRNA expressions of FTH1, SLC7A11, SLC3A2, GPX4, SLC40A1, and HSPB1 after TM4SF1 inhibition in SKOV3 and OVCAR8 cells. *p < 0.05; **p < 0.01; ***p < 0.001. Data are presented as the means ± SD. *p < 0.05, **p < 0.01, and ***p < 0.001 vs. shNC group; ^#^ *p < 0.05*, ^*##*^ *p < 0.01*, and ^*###*^ *p < 0.001* vs. shTM4SF1 group.

Abnormal iron and lipid ROS levels are typical characteristics of ferroptosis. Our previous research has suggested that PARPi promotes ferroptosis by repressing SLC7A11 in BRCA-proficient ovarian cancer (Hong et al., 2021). In addition, TM4SF1 has been reported to regulate ROS metabolism via the PPARγ–SIRT1 feedback loop (Cao et al., 2018). Therefore, we hypothesized that niraparib promoted ferroptosis by downregulating TM4SF1 expression. The immunofluorescence assay showed that the density of Fe^2+^ and lipid ROS was markedly increased following TM4SF1 knockdown. In addition, the ferroptosis inhibitor Fer-1 could rescue the increased Fe^2+^ and lipid ROS levels caused by TM4SF1 knockdown (Figures 5A,B). Morphological characteristics, such as a decrease in mitochondrial volume and an increase in membrane density, are prominent pathological hallmarks of ferroptosis. Thereafter, TEM was performed to clarify the association between TM4SF1 expression and ferroptosis. As shown in Figure 5C, TM4SF1 inhibition induced an increase in membrane volume and a decrease in mitochondrial volume in SKOV3 and OVCAR8 cell lines. Additionally, our results suggested that TM4SF1 knockdown inhibited cell proliferation partly by promoting ferroptosis (Figure 5D). To identify the specific molecular mechanism of TM4SF1 in suppressing ferroptosis, we first analyzed the correlation between TM4SF1 expression and ferroptosis-associated genes using the TCGA dataset. Our results showed that TM4SF1 was positively correlated with ferroptosis-related drivers (such as FTH1, GPX4, SLC40A1, and HSPB1) and negatively correlated with ferroptosis-related suppressors (such as ALOX15, BECN1, and SIRT1) (Figure 5E). Next, we measured the mRNA expression of ferroptosis-related biomarkers, including FTH1, SLC7A11, SLC3A2, GPX4, SLC40A1, and HSPB1. Our results revealed that TM4SF1 inhibition significantly downregulated their expression in SKVO3 and OVCAR8 cell lines (Figure 5F). All these results indicated that TM4SF1 might inhibit ferroptosis in BRCAwt OC cells.

## Discussion

According to clinical trials, the application of PARPi maintenance therapy has shown significant benefits for patients with HRD or BRCAm (DiSilvestro et al., 2023; Ray-Coquard et al., 2023). However, the QUADRA and PRIMA trials revealed that niraparib prolonged PFS in patients, regardless of BRCA or HRD status (Moore et al., 2019; González-Martín et al., 2023). This suggests that niraparib may exert additional anticancer effects beyond “synthetic lethality” in BRCAwt OC. DNA 6mA modification represents a vital epigenetic modification, and its abnormal status is implicated in human cancers and other diseases [13, 15]. In addition, multiple lines of evidence have revealed that 6mA is dynamically regulated following stress, and elevated 6mA modification might serve as an epigenetic signal for stress adaptation (Yao et al., 2017; Zhang et al., 2018; Otto, 2019; Hao et al., 2020). Recently, it has been reported that PARPi treatment can reduce the modification levels of 5-methylcytosine (5mC) and 5-hydroxymethylcytosine (5hmC), thereby potentially contributing to stress resilience (Cramer et al., 2019). However, the influence of niraparib on 6mA modification was unknown.

In our study, we found that the 6mA modification level in OC cells SKOV3 and OVCAR8 was significantly decreased after niraparib treatment. The demethylases ALKBH1 and ALKBH4 are responsible for DNA 6mA demethylation, while N6AMT1 and METTL4 are responsible for DNA 6mA methylation (Zhang et al., 2020). To explore the mechanism of niraparib-mediated downregulation of 6mA modification, we further measured the expression of these key demethylases and methylases. Surprisingly, niraparib treatment induced ALKBH1 expression but had no effect on the expressions of N6AMT1, METTL4, and ALKBH4. Additionally, low expression of ALKBH1 is marginally associated with poor prognosis in OC. Accumulating evidence has demonstrated that 6mA modification plays crucial roles in regulating gene transcriptional expression. Subsequently, we identified that ALKBH1 inhibited TM4SF1 expression by binding to its promoter and regulating its transcription. Niraparib downregulated the expression of TM4SF1 through ALKBH1-mediated 6mA modification. In addition, we demonstrated that TM4SF1, defined as a tumor-associated antigen, promoted tumor prognosis in OC *in vitro* and *in vivo*. Importantly, we demonstrated that TM4SF1 silencing induced ferroptosis by inhibiting the expression of ferroptosis-related driver genes.

Despite these promising results, our study has several limitations. First, the functions of ALKBH1-mediated 6mA vary across different cancers. For example, ALKBH1 knockdown resulted in an enhanced tumor malignancy in tongue squamous cell carcinoma (TSCC) (Xi et al., 2022), whereas it facilitated tumorigenesis in gastric cancer by inhibiting NRF1–AMPK signaling (Wang et al., 2023). The association between ALKBH1 and tumor prognosis in OC was assessed using bioinformatics analysis in our study. However, these results were not validated in our own patients. Additionally, the underlying mechanisms of how niraparib downregulates ALKBH1 expression remain unclear. In addition to catalyzing the attachment of ADP ribose units to target proteins, PARP1 also participates in transcriptional regulation (Kraus and Lis, 2003). Therefore, PARP1 might mediate the regulation of ALKBH1 by niraparib. Finally, future studies should also explore whether these findings extend to BRCAm models.

## Conclusion

**FIGURE 6:**
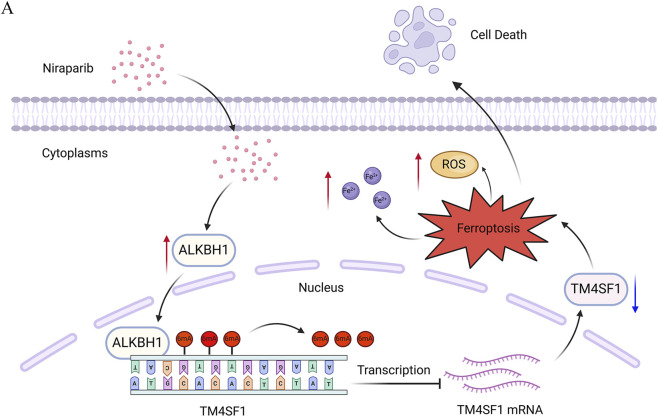
Schematic diagram of the regulative mechanism of niraparib in promoting ferroptosis by inhibiting TM4SF1 expression through ALKBH1-mediated 6mA modification in BRCA wild-type ovarian cancer.

In summary, our study demonstrated that niraparib exerts its antitumor effect and promotes ferroptosis by decreasing TM4SF1 expression through ALKBH1-mediated 6mA modification in BRCAwt OC (Figure 6). This provides novel insights into the antitumor effects of niraparib in BRCAwt OC and highlights the importance of epigenetic regulation in cancer therapy.

## Data Availability

The original contributions presented in the study are publicly available. This data can be found here: https://www.ncbi.nlm.nih.gov/sra/PRJNA1469481; https://www.ncbi.nlm.nih.gov/sra/PRJNA1470923.
