# Supplementary material for: Niraparib promotes ferroptosis by inhibiting TM4SF1 expression through ALKBH1-mediated 6mA modification in BRCA wild-type ovarian cancer
Source: Front Pharmacol. 2026 Jun 1;17:1706364. doi: 10.3389/fphar.2026.1706364 (PMC13266101; doi:10.3389/fphar.2026.1706364)
Supplement: Supplementary file 3 [file Table1.docx]

**Supplement table S1**. A list of the Primers sequence used in the study.

| Gene name | Primer sequence |
| --- | --- |
| GAPDH | Forward: GGAGCGAGATCCCTCCAAAAT |
|  | Reverse: GGCTGTTGTCATACTTCTCATGG |
| N6AMT1 | Forward: GCAGGGGAGAACTTCGCTAC |
|  | Reverse: CAGCGCGTTCAAAAGCAGAAA |
| ALKBH1 | Forward: AAACTTTTCCGCTTCTACCGTC |
|  | Reverse: TTTGAGTCCATAGGCTTGCCA |
| ALKBH4 | Forward: GTCCTTCGGGAATGCGGTT |
|  | Reverse: AATGAAACGGTATGTTTTCGCTG |
| METTL4 | Forward: TCTGTGGTACACCAGTTGTCA |
|  | Reverse: CCTTTTTACGGCAACAAGGTTCA |
| CCT6B | Forward: TCTCACTGTAGATTGCTTGGGA |
|  | Reverse: TGATAGCACGAAGTCCATCTCT |
| SRGAP3 | Forward: CCGCCGGAAAGCTGAGATT |
|  | Reverse: GCTGCGGATTTTGGAGGAGA |
| RAPSN | Forward: GCAGGTGTGGACAAAGGTG |
|  | Reverse: CCAGGTTCAGGTAGCTCTCC |
| NEUROD2 | Forward: TGCTACTCCAAGACGCAGAAG |
|  | Reverse: CACGTAGGACACTAGGTCTGG |
| TM4SF1 | Forward: TGCATCGGACATTCTCTGGTG |
|  | Reverse: GTTCCAGCCCAATGAAGACAA |
| SARNP | Forward: AATGTCTTGCTCGTGGTTTGG |
|  | Reverse: AGCTCAATGGGCTTTGTTTCT |
| SLC26A7 | Forward: AGAAGGCGACTGCCCATTTT |
|  | Reverse: ACTGCCAACATTATCCCAGACA |
| EFCAB6 | Forward: AGACTGGACTGGTTCGACC |
|  | Reverse: GGCTTGTTGATTCTCCAACGAG |
| KCNB1 | Forward: ACTCTGGCGTACCCTGGAC |
|  | Reverse: GTCGTCGAGGCTGTAGTCATC |
| IGF2BP1 | Forward: GCGGCCAGTTCTTGGTCAA |
|  | Reverse: TTGGGCACCGAATGTTCAATC |
