# Supplementary material for: Niraparib promotes ferroptosis by inhibiting TM4SF1 expression through ALKBH1-mediated 6mA modification in BRCA wild-type ovarian cancer
Source: Front Pharmacol. 2026 Jun 1;17:1706364. doi: 10.3389/fphar.2026.1706364 (PMC13266101; doi:10.3389/fphar.2026.1706364)
Supplement: Supplementary file 4 [file Table4.docx]

**Supplement table S4**. Intersected genes in RNA-seq and 6mA-IP-seq in SKOV3 cell line.

| **Gene_ID** | **6mA-IP-seq** | | | **RNA-seq** | | |
| --- | --- | --- | --- | --- | --- | --- |
|  | Position | FC | *P* adj | Up/down | logFC | *P* adj |
| CCT6B | Intron | 4.36 | 7.01E-08 | up | 1.096039475 | 0.003187367 |
| SRGAP3 | Intron | 3.94 | 5.64E-08 | up | 2.058495064 | 6.44E-26 |
| RAPSN | Intron | 2.73 | 5.67E-05 | Up | 1.590774873 | 4.86E-05 |
| NEUROD2 | 3' UTR | 2.64 | 3.63E-06 | Up | 1.729659257 | 0.002631861 |
| TM4SF1 | Promoter | 3.98 | 1.90E-06 | Down | -1.663908243 | 3.94E-27 |
| SARNP | Intron | 2.53 | 4.32E-05 | Up | 1.397202835 | 0.044893203 |
| SLC26A7 | Intron | 2.65 | 3.39E-05 | Up | 1.101285587 | 0.002101294 |
| EFCAB6 | Intron | 3.16 | 2.72E-05 | Up | 1.45243865 | 3.85E-06 |
| KCNB1 | Intron | 2.82 | 9.21E-06 | Up | 1.557928034 | 0.001315355 |
| IGF2BP1 | Exon | 2.82 | 8.46E-06 | Down | -1.952051532 | 0.025550562 |
