# Supplementary material for: Niraparib promotes ferroptosis by inhibiting TM4SF1 expression through ALKBH1-mediated 6mA modification in BRCA wild-type ovarian cancer
Source: Front Pharmacol. 2026 Jun 1;17:1706364. doi: 10.3389/fphar.2026.1706364 (PMC13266101; doi:10.3389/fphar.2026.1706364)
Supplement: Supplementary file 5 [file DataSheet1.docx]

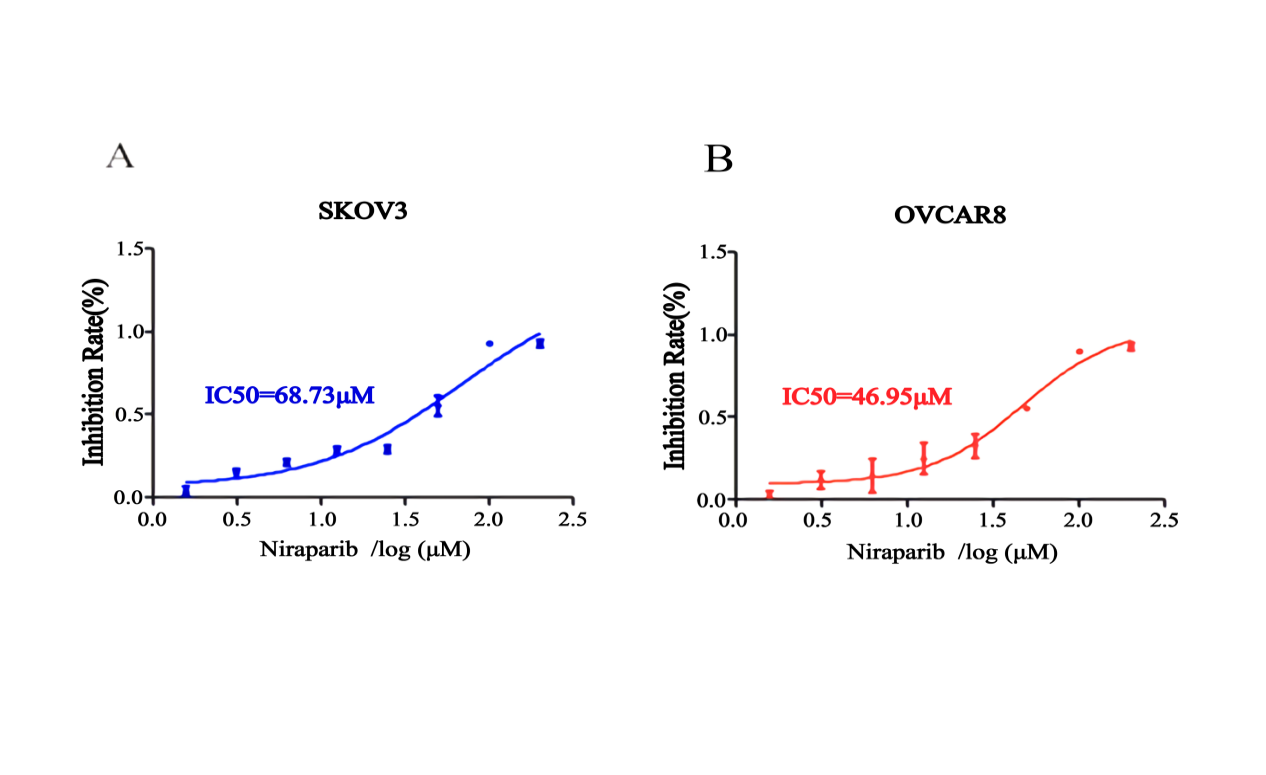


**Figure S1.** (A, B) IC50 values of niraparib in SKOV3 and OVCAR8 cells.


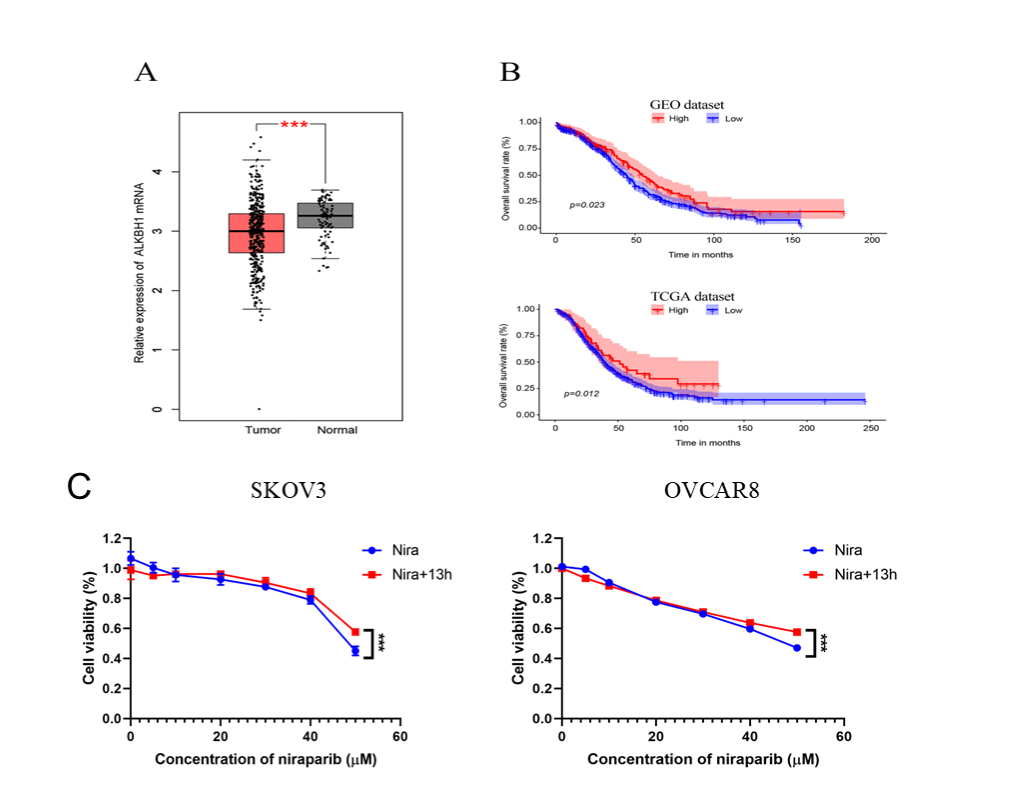


**Figure S2.** ALKBH1 was downregulated in ovarian cancer and low ALKBH1 expression was significantly associated with poor prognosis in ovarian cancer. (**A**) Data from the TCGA and GTEx databases showed that ALKBH1 was downregulated in ovarian cancer tissues compared to ovarian normal tissues. (B) Kaplan-Meier survival analysis of ALKBH1 expression in the GEO and TCGA databases.


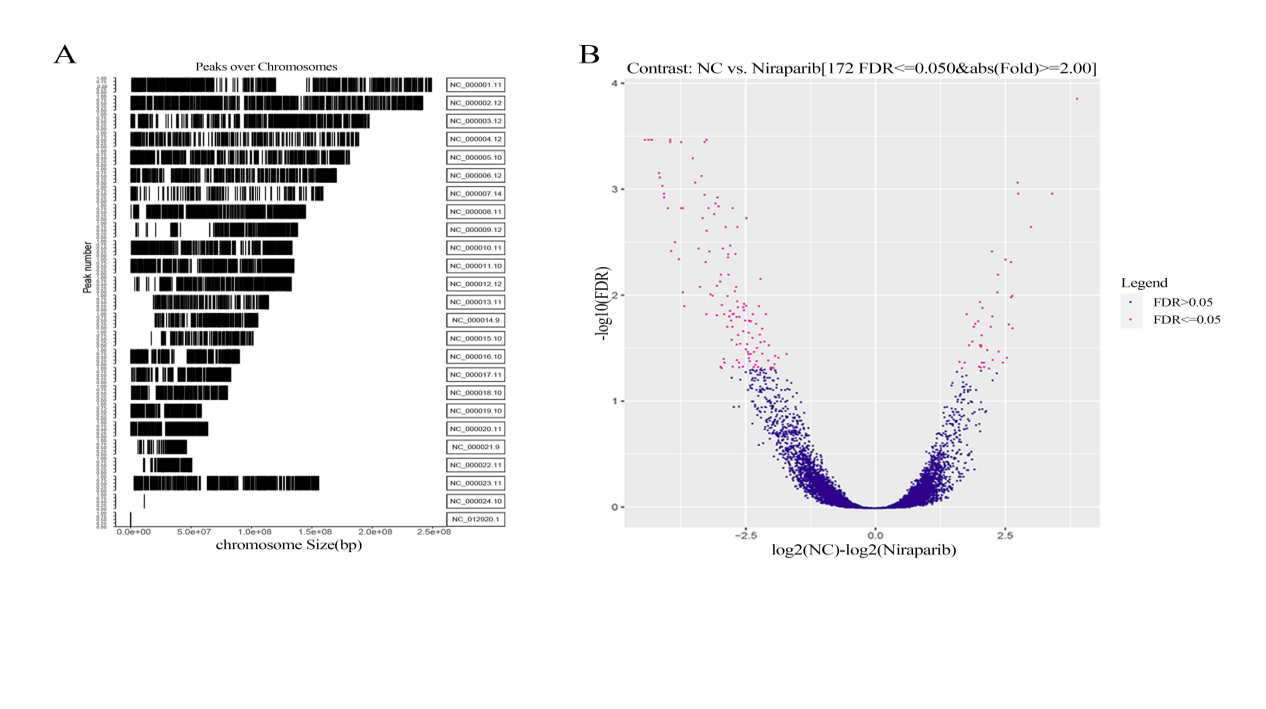


**Figure S3.** (A) Distribution of 6mA modifications on the chromosome. (B) Visualization of differential genes obtained by differential analysis.


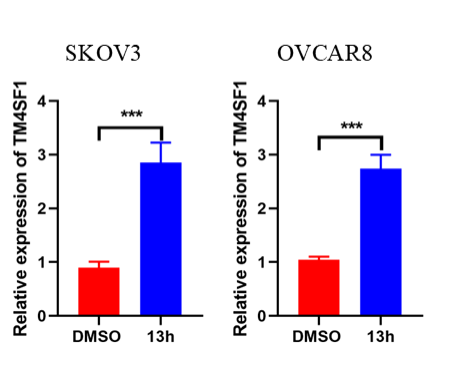


**Figure S4.** The compound 13h significantly increased the expression of *TM4SF1 mRNA*.


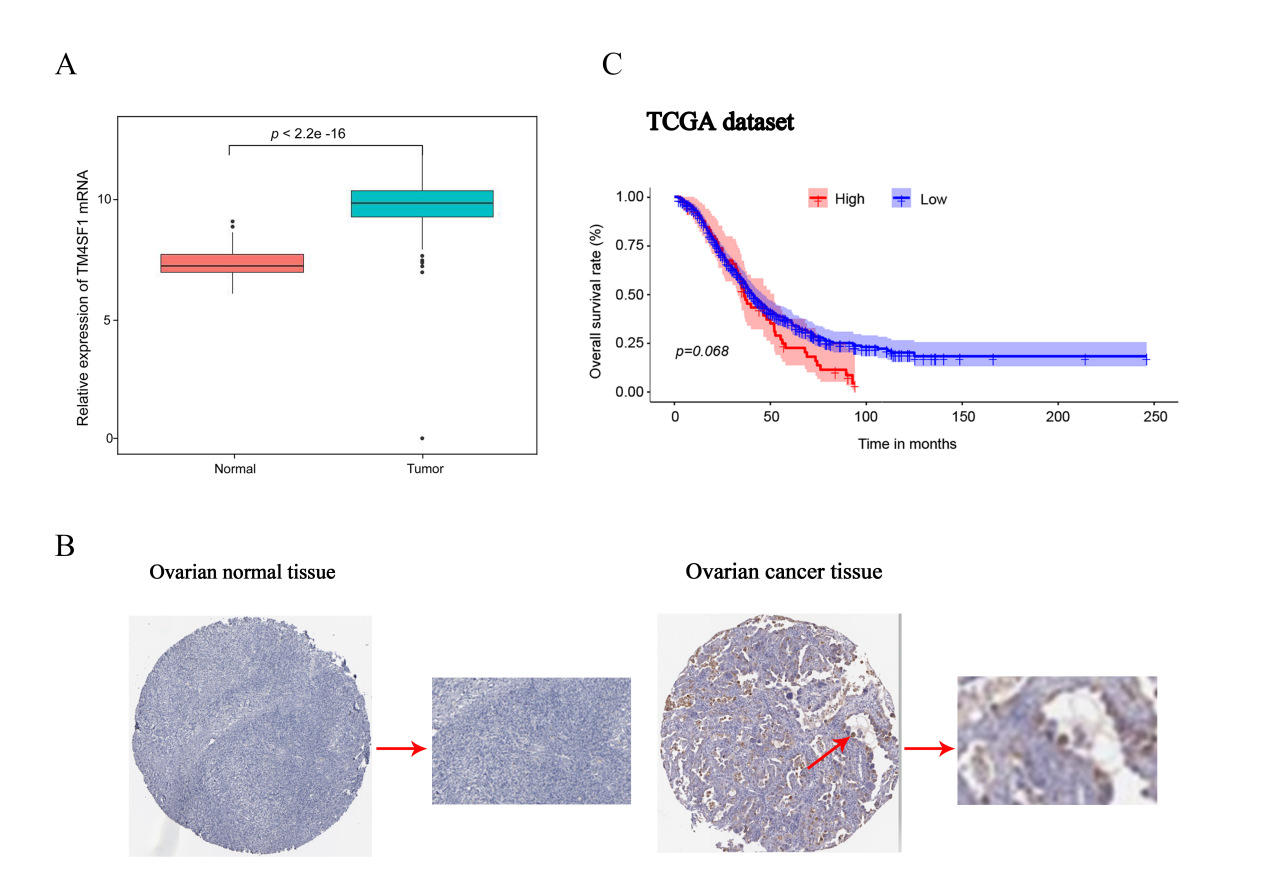


**Figure S5.** TMSF1 was upregulated and high TM4SF1 expression was significantly associated with poor prognosis in ovarian cancer. (**A**) Data from the TCGA and GTEx databases showed that TM4SF1 was upregulated in ovarian cancer tissues compared to ovary normal tissues. (**B**) Immunohistochemical staining for TM4SF1 in ovarian cancer tissues and ovarian normal tissues (**C**) Kaplan-Meier survival analysis of TM4SF1 expression in the TCGA database.
